# Supplementary material for: Advanced EPI-X4 Derivatives Covalently Bind Human Serum Albumin Resulting in Prolonged Plasma Stability
Source: Int J Mol Sci. 2022 Nov 30;23(23):15029. doi: 10.3390/ijms232315029 (PMC9735595; doi:10.3390/ijms232315029)
Supplement: Supplementary file 1 [file ijms-23-15029-s001.zip › ijms-1980801-supplementary.pdf]

Supplement

Advanced EPI-X4 Derivatives Covalently Bind Human Serum Albumin Resulting in Prolonged Plasma Stability

Armando Rodríguez-Alfonso [1,2], Astrid Heck [3], Yasser Bruno Ruiz-Blanco [4], Andrea Gilg [5], Ludger Ständker [1], Seah Ling Kuan [3], Tanja Weil [3], Elsa Sanchez-Garcia [4], Sebastian Wiese [2], Jan Münch [1,5,*] and Mirja Harms [5,*]

[1] Core Facility Functional Peptidomics, Ulm University Medical Center, 89081 Ulm, Germany
[2] Core Unit Mass Spectrometry and Proteomics, Ulm University Medical Center, 89081 Ulm, Germany
[3] Max Planck Institute for Polymer Research, 55128 Mainz, Germany
[4] Center of Medical Biotechnology, University of Duisburg-Essen, 47057 Duisburg, Germany
[5] Institute of Molecular Virology, Ulm University Medical Center, 89081 Ulm, Germany

* Correspondence: jan.muench@uni-ulm.de (J.M.); mirja.harms@uni-ulm.de (M.H.)

a

b

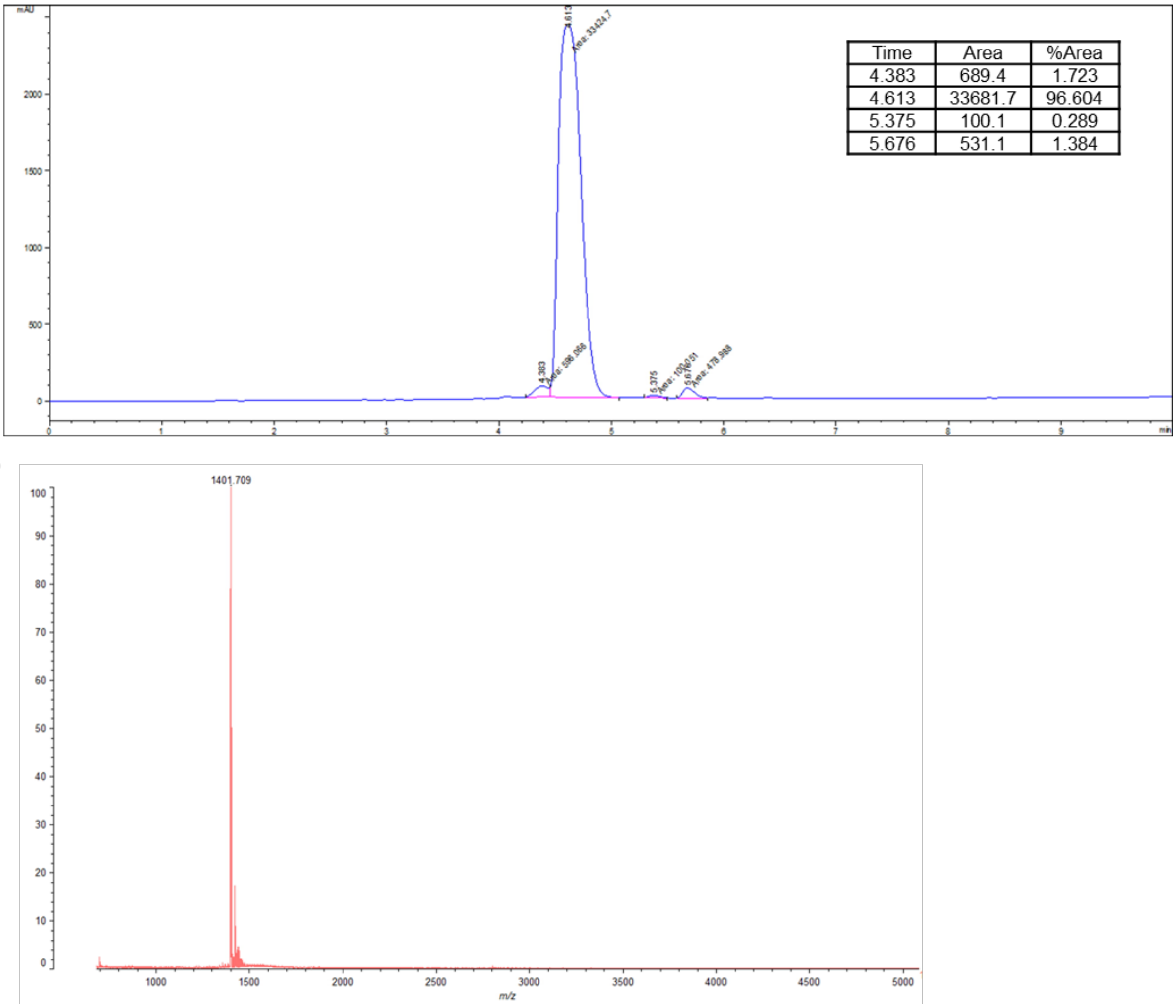

Time	Area	%Area
4.383	689.4	1.723
4.613	33681.7	96.604
5.375	100.1	0.289
5.676	531.1	1.384

Figure S1. (a) RPC18-HPLC analysis of synthetic WSC02 (IVRWSKKVPCVS). WSC02 was applied into a Biobasic 18 column (Thermo Fischer Scientific, USA) of dimensions 2.1 x 100 mm, 5 µm. The peptide was eluted using a gradient of acetonitrile from 5% to 45% in 10 min, at a flow rate of 0.5 mL/min. Elution was monitored by online UV detection at 214 nm. WSC02 purity was 96.6%. (b) The mass spectrometry analysis of synthetic WSC02 was performed with an AXIMA Confidence MALDI-TOF MS system (Shimadzu, Japan). The m/z value of 1401.709 very closely matched the theoretical monoisotopic m/z value for this peptide (1401.792).

a

b

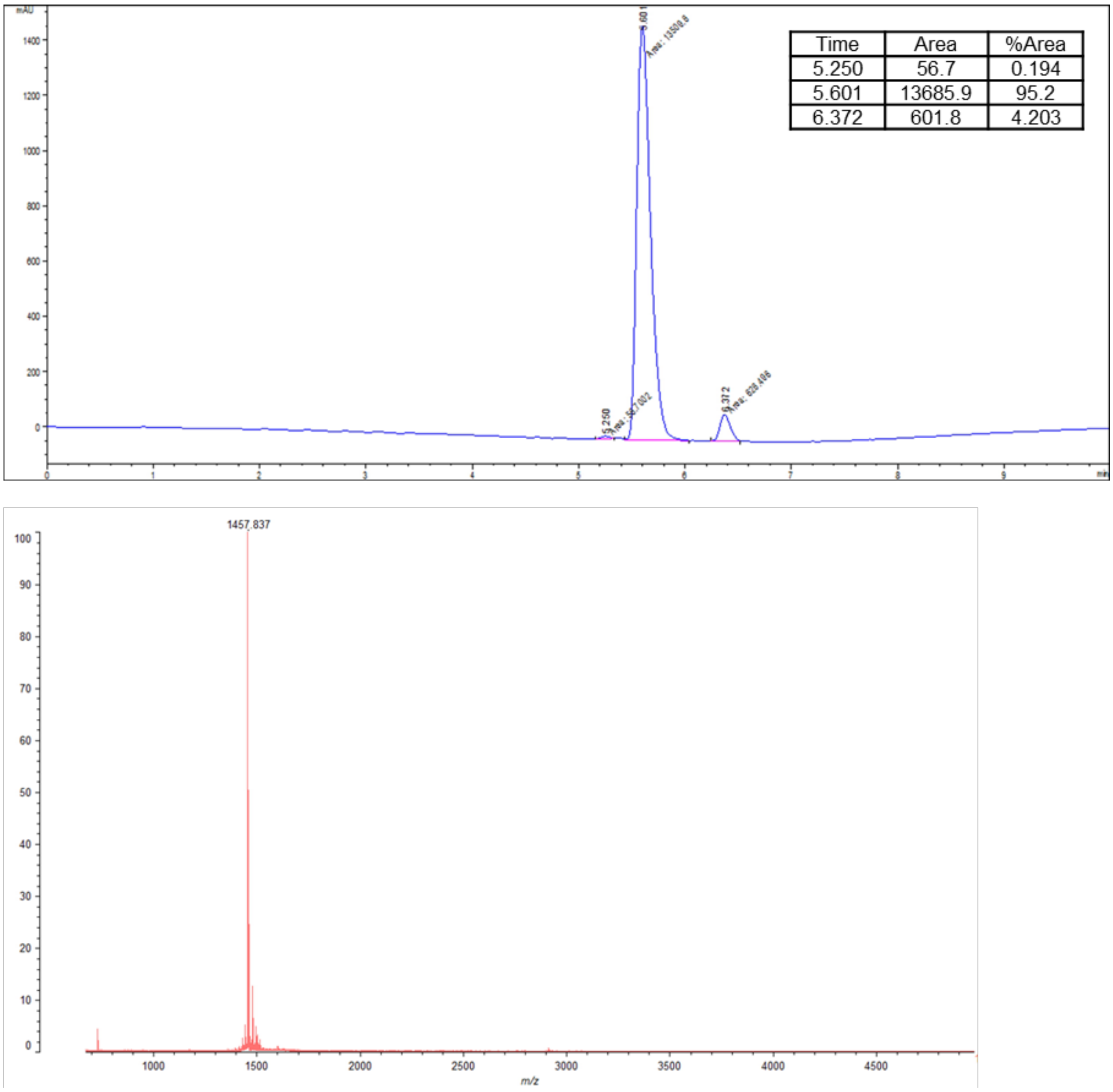

Figure S2. (a) RPC18-HPLC analysis of synthetic JM#21 (ILRWSRKLPCVS). JM#21 was applied into a Biobasic 18 column (Thermo Fischer Scientific, USA) of dimensions 2.1 x 100 mm, 5 µm. The peptide was eluted using a gradient of acetonitrile from 5% to 45% in 10 min, at a flow rate of 0.5 mL/min. Elution was monitored by online UV detection at 214 nm. JM#21 purity was 95.2%. (b) The mass spectrometry analysis of synthetic JM#21 was performed with an AXIMA Confidence MALDI-TOF MS system (Shimadzu, Japan). The m/z value of 1457.837 very closely matched the theoretical monoisotopic m/z value for this peptide (1457.830).

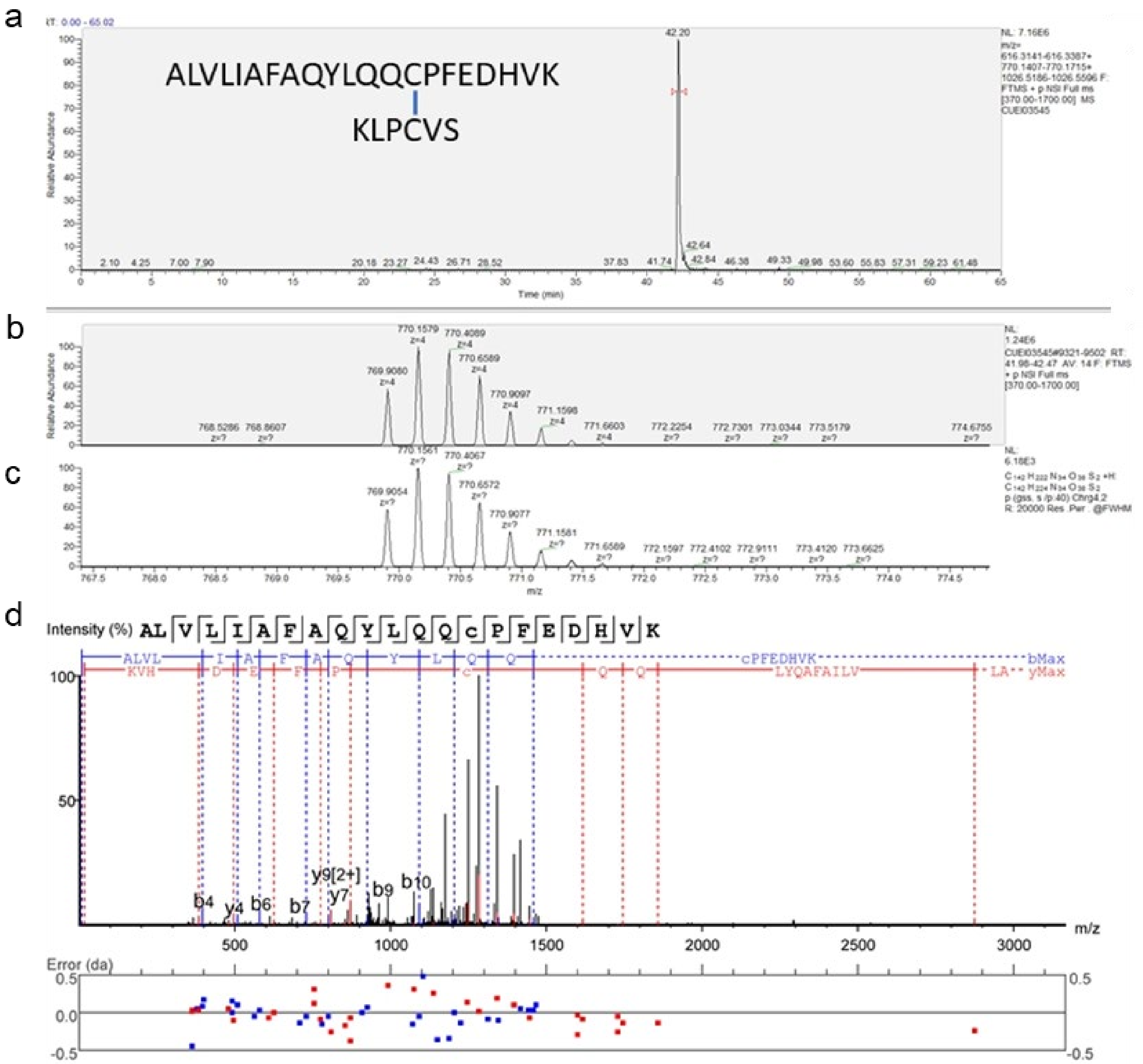

Figure S3. Identification of the albumin fragment containing Cys34 bound to JM#21 via disulfide bridge, after digestion with Trypsin and MS analysis. (a) Extracted ion chromatogram to identify the fragment of albumin (ALVLIAFAQYLQQCPFEDHVK), expected to be released from the digestion with Trypsin, bound by disulfide bridge to a fragment (KLPCVS, one missing cleavage, +643.33 Da) of JM#21. (b) Experimental isotopic distribution (z=4) of the molecule, which perfectly matches the (c) theoretical isotopic distribution (z=4). d) MS/MS spectrum of the fragments bound by a disulfide bridge.

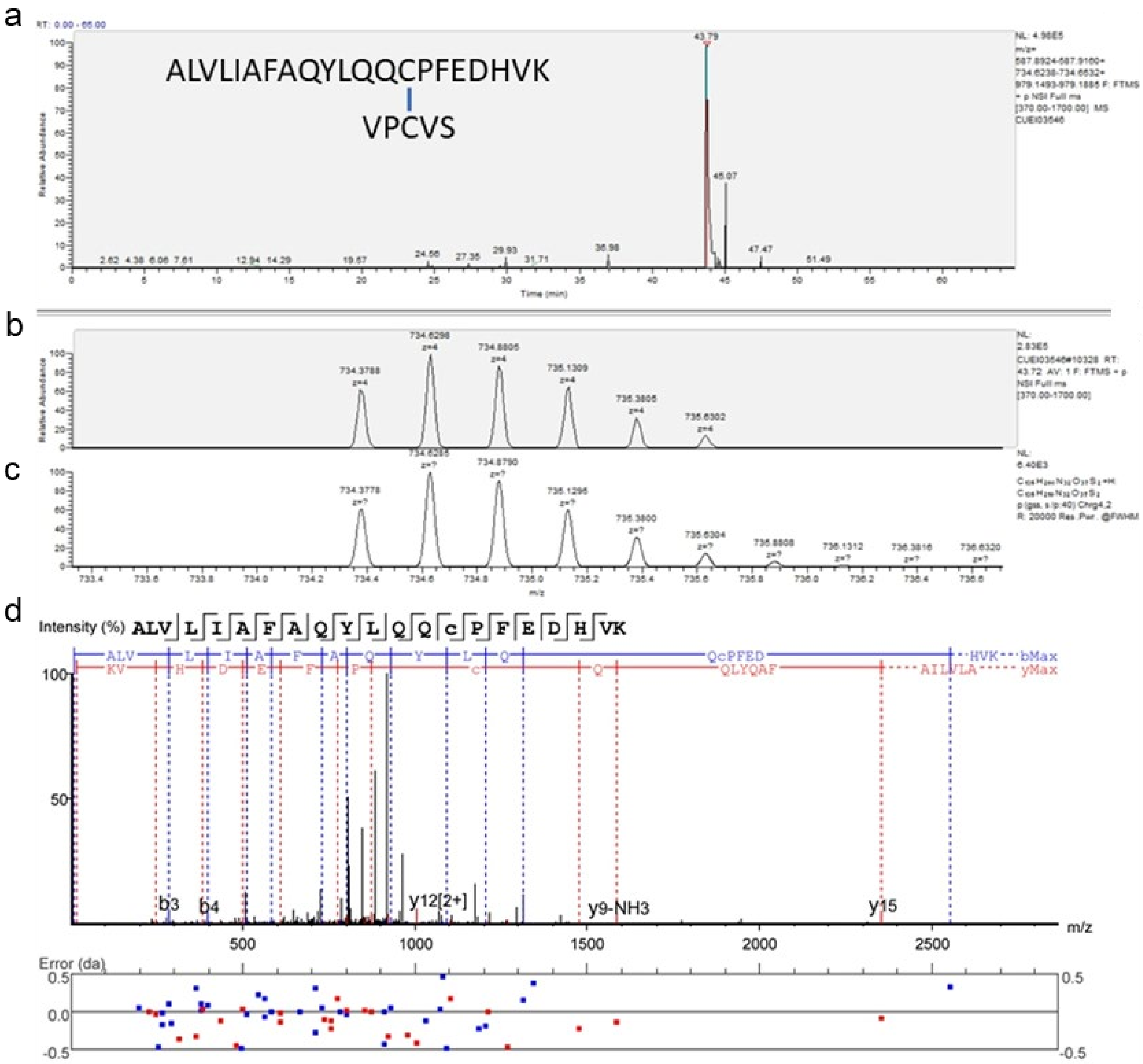

Figure S4. Identification of the albumin fragment containing Cys34 bound to WSC02 via disulfide bridge, after digestion with Trypsin and MS analysis. (a) Extracted ion chromatogram to identify the fragment of albumin (ALVLIAFAQYLQQCPFEDHVK), expected to be released from the digestion with Trypsin, bound by disulfide bridge to a fragment (VPCVS, +501.23 Da) of WSC02. (b) Experimental isotopic distribution (z=4) of the molecule, which perfectly matches the (c) theoretical isotopic distribution (z=4). (d) MS/MS spectrum of the fragments bound by a disulfide bridge.

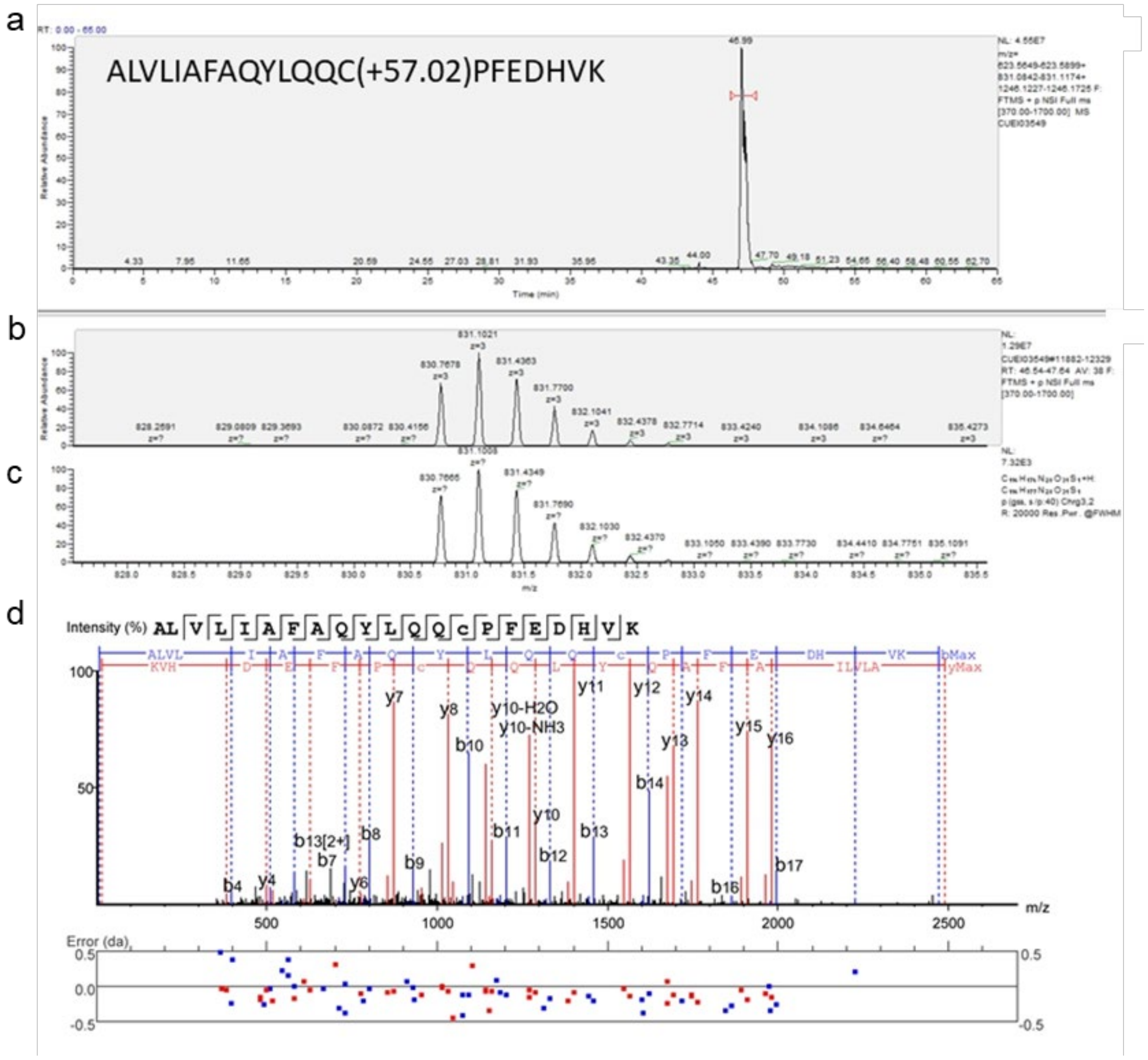

Figure S5. Carbamidomethylated HAS does not bind peptides via S-S. (a) Extracted ion chromatogram to identify the fragment of albumin (ALVLIAFAQYLQQCPFEDHVK), expected to be released from the digestion with Trypsin, after carbamidomethylation (+57.02 Da). (b) Experimental isotopic distribution (z=3) of the molecule, which perfectly matches the (c) theoretical isotopic distribution (z=3). (d) MS/MS spectrum of the carbamidomethylated albumin fragment.

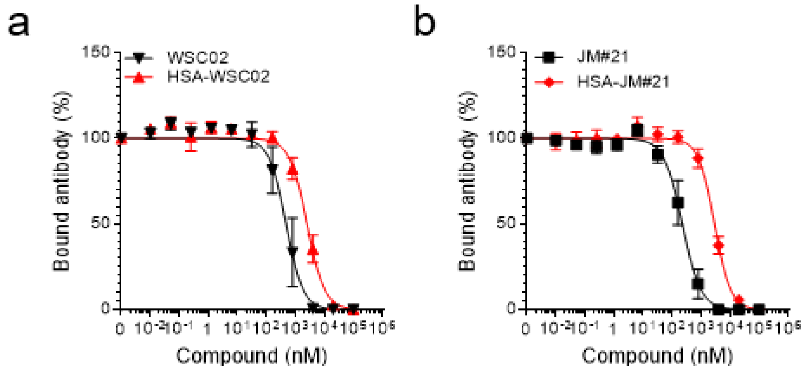

Figure S6. EPI-X4 WSC02 and JM#21 covalently bound to HSA are biologically active. Activity of WSC02 (a) or JM#21 (b) conjugates or unconjugated peptides were analyzed using the antibody competition assay. Data derived from 3 individual experiments ± SEM are shown.
